# Correction: Lacy et al. Human Rabies Treatment—From Palliation to Promise. *Viruses* 2024, *16*, 160

**DOI:** 10.3390/v16020264

**Published:** 2024-02-07

**Authors:** Marian Lacy, Nonthapan Phasuk, Stephen J. Scholand

**Affiliations:** 1 College of Medicine, University of Arizona, Tucson, AZ 85721, USA; mlacy@arizona.edu; 2 School of Medicine, Walailak University, Nakhon Si Thammarat 80160, Thailand; nonthapan.ph@wu.ac.th

## Revise Informed Consent Statement 

In the original publication [1], there was an error of omission in the Informed Consent Statement Section: ”Not applicable”. The correct Informed Consent Statement appears below: 

“Informed consent was obtained from all subjects (one patient seen in Figure 1) involved in the study. Written informed consent has been obtained from the patient (and his parents) in Figure 1 to publish this paper.”

The authors state that the scientific conclusions are unaffected. This correction was approved by the Academic Editor. The original publication has also been updated.
